# Strength Exercise Beyond Walking: Risk Factor for Sarcopenia in Early Older Adults (65–75 Years)

**DOI:** 10.1093/geroni/igaf122.2752

**Published:** 2025-12-31

**Authors:** Layoung Kim, Gwang Suk Kim, Sooyoung Kwon

**Affiliations:** The University of Suwon, Hwaseong, Republic of Korea; Yonsei University College of Nursing, Seoul, Republic of Korea; Graduate School of Yonsei University, Seoul, Republic of Korea

## Abstract

Sarcopenia is defined as ‘age-related loss of muscle mass, plus low muscle strength, and/or low physical performance.’ In early older adults, it is rarely diagnosed based on physical performance, underscoring the importance of early intervention strategies. This study aimed to identify risk factors associated with sarcopenia in adults aged 65–75 years. Using the 2023 Korean National Health and Nutrition Examination Survey data, participants were categorized into three groups: sarcopenia (low muscle mass and low strength), sarcopenia risk (low muscle mass or low strength, excluding sarcopenia), and robust. The classification was based on appendicular skeletal muscle mass and handgrip strength, following the 2019 Asian Working Group for Sarcopenia criteria. Demographic characteristics and physical and psychological health-related factors were included as independent variables. Multinomial logistic regression was used to estimate odds ratios (OR) for factors associated with sarcopenia and sarcopenia risk. Among the 915 participants, the prevalence rates of sarcopenia and sarcopenia risk were 5.6% and 27.6%, respectively. Prevalence increased with age and lower BMI. Multivariate multinomial logistic regression analysis revealed that compared to the robust group, both sarcopenia and sarcopenia risk were significantly associated with not engaging in strength exercises at least once per week (OR = 2.598, OR = 1.858) and experiencing higher usual stress levels (OR = 3.034, OR = 1.710). However, not meeting the WHO’s recommended 2.5 hours of weekly moderate-intensity aerobic physical activity was not significantly associated with sarcopenia or sarcopenia risk. This finding suggests that strength exercises beyond walking, should be emphasized for early older adults.

